# Mortality rate and predictors of COVID-19 inpatients in Ethiopia: a systematic review and meta-analysis

**DOI:** 10.3389/fmed.2023.1213077

**Published:** 2023-10-20

**Authors:** Molla Yigzaw Birhanu, Selamawit Shita Jemberie

**Affiliations:** ^1^Department of Public Health, College of Medicine and Health Sciences, Debre Markos University, Debre Markos, Ethiopia; ^2^Department of Midwifery, College of Medicine and Health Sciences, Debre Markos University, Debre Markos, Ethiopia

**Keywords:** mortality, inpatient, predictors, COVID-19 patients, incidence, Ethiopia

## Abstract

**Introduction:**

The severe acute respiratory syndrome coronavirus 2 (SARS-CoV-2) is an extremely rare virus that devastates the economy and claims human lives. Despite countries' urgent and tenacious public health responses to the COVID-19 pandemic, the disease is killing a large number of people. The results of prior studies have not been used by policymakers and programmers due to the presence of conflicting results. As a result, this study was conducted to fill the knowledge gap and develop a research agenda.

**Objective:**

This study aimed to assess the mortality rate and predictors of COVID-19 hospitalized patients in Ethiopia.

**Methods:**

Electronic databases were searched to find articles that were conducted using a retrospective cohort study design and published in English up to 2022. The data were extracted using a Microsoft Excel spreadsheet and exported to Stata^TM^ version 17.0 for further analysis. The presence of heterogeneity was assessed and presented using a forest plot. The subgroup analysis, meta-regression, and publication bias were computed to identify the source of heterogeneity. The pool COVID-19 mortality rate and its predictors were calculated and identified using the random effects meta-analysis model, respectively. The significant predictors identified were reported using a relative risk ratio and 95% confidence interval (CI).

**Results:**

Seven studies with 31,498 participants were included. The pooled mortality rate of COVID-19 was 9.13 (95% CI: 5.38, 12.88) per 1,000 person-days of mortality-free observation. Those study participants who had chronic kidney disease had 2.29 (95% CI: 1.14, 4.60) times higher chance of experiencing mortality than their corresponding counterparts, diabetics had 2.14 (95% CI: 1.22, 3.76), HIV patients had 2.98 (95% CI: 1.26, 7.03), hypertensive patients had 1.63 (95% CI: 1.43, 1.85), and smoker had 2.35 (95% CI: 1.48, 3.73).

**Conclusion:**

COVID-19 mortality rate was high to tackle the epidemic of the disease in Ethiopia. COVID-19 patients with chronic renal disease, diabetes, hypertension, smoking, and HIV were the significant predictors of mortality among COVID-19 patients in Ethiopia. COVID-19 patients with chronic diseases and comorbidities need special attention, close follow-up, and care from all stakeholders.

## Introduction

The newly emerged coronavirus disease 2019 (COVID-19), caused by the severe acute respiratory syndrome coronavirus-2 (SARS-CoV-2), has rapidly spread throughout the world with varying clinical characteristics (1). The COVID-19 pandemic remains an unpredictable debacle with symptoms ranging from asymptomatic infection to a fulminant systemic inflammatory syndrome unleashed by a cytokine storm (2). It has the potential to attack all human body systems (3). It is associated with significant global morbidity and in-hospital mortality rates, and due to its highly infectious nature, it will continue to spread (4). During the pandemic, high healthcare resources were utilized for caring for critical cases, which imposed enormous pressure on healthcare systems worldwide (5, 6). More recent COVID-19 data show that severe disease conditions, disease progression, and mortality risk are heavily influenced by underlying comorbidities and specific complications, which result in increased risk and a poor prognosis (7, 8). When compared to an initial infection with severe acute respiratory distress syndrome coronavirus 2, pre-existing comorbidities and short-term complications can result in more severe outcomes, such as exacerbations and death (SARS-CoV-2) (9, 10). Identifying these prognosis factors in a clinical setting is important in managing COVID-19 inpatients and contributing to future clinical decisions to resolve health threats in healthcare systems (9, 11, 12). Even though there are studies conducted on the incidence and predictors of COVID-19 mortality in Ethiopia, there is a lack of homogeneity across studies. Hence, it may be difficult to use them for policy formulation and intervention as well as by healthcare professionals and programmers. To provide an appropriate public health and clinical response, we sought to determine the COVID-19 mortality rate and identify predictors in Ethiopia.

**Research question**: What is the COVID-19 mortality rate and its predictors in Ethiopia?

**Condition:** COVID-19.

**Context:** Ethiopia.

**Study population:** COVID-19 patients.

## Methods

### Study area

Ethiopia is the Federal Democratic Republic with nine regional states (Afar, Amhara, Benishangul-Gumuz, Gambella, Harari, Oromia, Somali, Southern Nations Nationalities and People's Region, and Tigray) and two city administrations (Addis Ababa and Dire Dawa). The country has a total area of 1,100,000 km^2^ and is divided into zones, which are further subdivided into districts. Districts are further subdivided into kebeles, the lowest administrative divisions (13). Ethiopia, with a population of ~112 million people, is Africa's second-most populous country (56,010,000 female and 56,069,000 male individuals in 2019) (14).

### Data searching strategy from source

This review was reported using the Strengthening the Reporting of Observational Studies in Epidemiology (STROBE) guideline (15) (Supplementary material). The articles about our topic of interest were searched systematically from international and national electronic databases such as PubMed/MEDLINE, CINAHL, EMBASE, African Journals Online, Google Scholar, and ScienceDirect. In addition to the databases, articles were identified by searching the reference lists of eligible studies. Two authors (MYB and SSJ) searched independently. Endnote X9 was used to retrieve and manage studies searched out from different databases. An example of a search engine approach applied in the PubMed database searches is shown below: “(((((((COVID-19 infected [Text Word]) OR (COVID-19 patients [Text Word])) OR (COVID-19 patients [MeSH Terms])) AND (rate [Text Word])) OR (incidence (Text word)] OR (predictors (Text word)] OR (patient with COVID-19) [MeSH Terms])) AND (Ethiopia [MeSH Terms])) OR (Ethiopia [Text Word]).” The search strategy was performed from 10 to 30 March 2023.

### Eligibility criteria

The eligibility criteria were developed using the logic grid structure considering “Context, Condition, and Population (COCOPop).”

### Inclusion criteria

All cohort studies conducted on “incidence and predictors of COVID-19 mortality in Ethiopia” and published in English were included.

### Exclusion criteria

The titles of the articles that were similar to this study but different in the interest of outcomes (not reporting incidence and predictors) were excluded.

## Design: systematic review and meta-analysis

### Screening procedure

**Figure 1:**
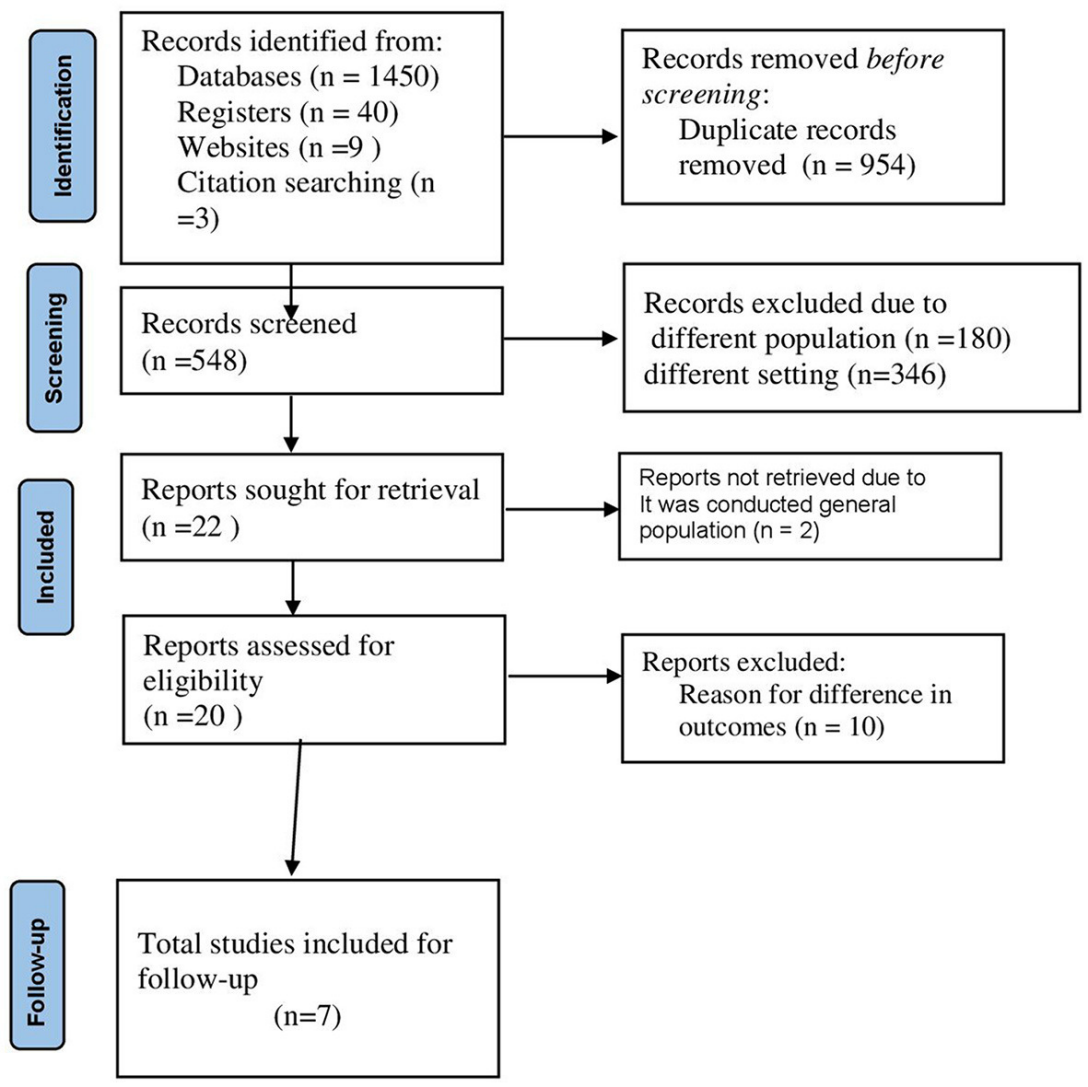
STROBE flow diagram for the included studies of COVID-19 mortality in Ethiopia.

Two authors (MYB and SSJ) screened all titles/abstracts of the searched articles independently. To assure the transparency and validity of the included articles, the screened results of the two authors were cross-checked, and the presence of conflicting results was discussed and fixed based on scientific merit. The authors who worked on the title and abstract screening also worked on the full-length screening independently and came to a consensus on the included articles (Figure 1).

### Quality assessment/risk of bias

The Newcastle-Ottawa Quality Assessment Scale for cohort study (16) was used to evaluate the quality of the included studies. This guideline contains selection (representativeness of the exposed cohort, selection of the non-exposed cohort, ascertainment of exposure, and demonstration that an outcome of interest was not present at the start of the study), comparability (based on the design or analysis controlled for confounders), and outcome (assessment of outcome, was follow-up long enough for outcomes to occur, and adequacy of follow-up of cohorts). The assessment tool guides that the articles that scored 3 or 4 stars in the selection domain AND 1 or 2 stars in the comparability domain AND 2 or 3 stars in the outcome/exposure domain were declared as “good quality,” 2 stars in selection domain AND 1 or 2 stars in comparability domain AND 2 or 3 stars in outcome/exposure domain were declared as “fair quality,” and 0 or 1 star in selection domain OR 0 stars in comparability domain OR 0 or 1 stars in outcome/exposure domain were declared as “poor quality” as a result, during the quality assessment of articles, all included articles were declared as having good quality.

### Data extraction

The data were extracted using the data extraction checklist prepared from the Microsoft Excel spreadsheet and adapted from the JBI data extraction spreadsheet. To ensure consistency, two authors (MYB and SSJ) extracted the data independently and cross-checked the quality and content of the data. The difference in the data extraction between the authors was solved through discussion. The extraction checklist has two sections, depending on the objectives. The data extracted for the first objective were region, publication year, sample size, follow-up period, first author name, sampling methods, follow-up period, incidence, and events during extraction. The data extraction checklist for predictors contains the hazard ratio, lower and upper confidence intervals, exposed and cases, exposed and non-cases, non-exposed, and non-cases of the predictors.

### Outcome variable and measures

In this study, two outcomes were measured. The primary outcome of interest was the pooled mortality rate of COVID-19 patients in Ethiopia. It was calculated by considering the incidence of the individual studies and the standard error of the incidence of COVID-19 mortality in Ethiopia. The second outcome of interest was the pooled predictors of COVID-19 mortality in Ethiopia, which were calculated by taking the logarithm of the relative risk and the standard error of the logarithm of the relative risk using a binary meta-regression model and reporting the relative risk with 95% confidence interval (CI).

### Data processing and analysis

The extracted data were exported to the Stata^TM^ version 17.0 software from a Microsoft Excel spreadsheet. The pooled mortality rate of COVID-19 was estimated using the Metaprop Stata command. The standard errors were calculated from the reported estimates and population denominators using a binomial distribution assumption. The presence of heterogeneity among studies was assessed using Cochran's Q test and quantified using the *I*^2^ statistics. Heterogeneity is classified as low (*I*^2^ values: 0–25), moderate (*I*^2^ values: 25–50), high (*I*^2^ values: 50–75), and very high (*I*^2^ values: ≥75) (17). To try to identify the source of heterogeneity, computing subgroup analysis, meta-regression, publication bias, and sensitivity analysis were used. To estimate the effect sizes, a random-effects model based on DerSimonian and Laird methods was used (18). The scatterplot in the funnel plot was assessed as the initial publishing bias evaluation, followed by Egger's linear regression test, which demonstrated that small studies had no effect on publication bias, and finally, trim and fill analysis tests were computed to obtain a conclusion (19). Finally, the findings were presented using texts, tables, and figures such as forest plots.

## Results

### Search results

A total of 630 studies were searched from electronic databases such as PubMed/MEDLINE, CINAHL, EMBASE, Google Scholar, and ScienceDirect, as well as organizational records, references, and websites. Approximately 560 articles were excluded due to duplication, 60 articles were excluded due to differences in study setting/context (17, 19–23), and 10 articles were excluded due to differences in outcome of interest (20, 24–26). Finally, seven retrospective cohort studies were included in this systematic review and meta-analysis.

### Characteristics of studies

In Ethiopia, approximately seven studies with 31,498 children were included and followed. These studies were conducted in the Amhara region (*n* = 3) and the Oromia region (*n* = 4). All of the included studies were conducted through a retrospective follow-up method. Studies with the smallest (288) and largest (28,533) sample sizes were conducted in the Amhara region. The included studies' follow-up periods ranged from 3 to 22 days, with 3,127 to 243,887 person-days of COVID-19 mortality-free observations (Table 1).

**Table 1:** The characteristics of the included studies.

**Author**	**Publication year**	**Study region**	**Mean sample size**	**Sample size**	**Mortality**	**PDO**	**ID/1,000**	**Follow up in days**	**Mean follow up in days**	**Study quality**
Galana Mamo Ayana et al. (27)	2021	Oromia	>494	531	101	6,235	16.2	6	< 7	Good
Belayneh Mengist et al. (28)	2022	Amhara	>494	552	29	6,155	4.7	12	>7	Good
Abdene Weya Kaso et al. (29)	2022	Oromia	<494	422	47	7,402	6.35	9	>7	Good
Tadesse Tolossa et al. (30)	2022	Oromia	<494	318	51	3,618	14.1	9	>7	Good
Anteneh Mengist Dessie et al. (31)	2022	Amhara	>494	28,533	2873	243,887	11.78	22	>7	Good
Ephrem Mannekulih Habtewold et al. (32)	2022	Oromia	>494	854	97	9,798	9.9	5	<7	Good
Fassikaw Kebede (33)	2022	Amhara	<494	288	58	3,127	1.8	3	<7	Good

PDO, person day free observation; FPD, follow –up period in day; RR, relative risk; ID, incidence density.

### The pooled COVID-19 mortality rate

The mortality rate of COVID-19 inpatients was 9.13 (95% CI: 5.38, 12.88) per 1,000 person-days of COVID-19 mortality-free observations. When we looked at it by region, the Oromia region had the highest COVID-19 mortality rate (ES: 11.48 (95% CI: 7.31, 15.64), and the Amhara region had the lowest (ES: 6.12 (95% CI: −0.89, 13.14).

### Subgroup meta-analysis

**Figure 2:**
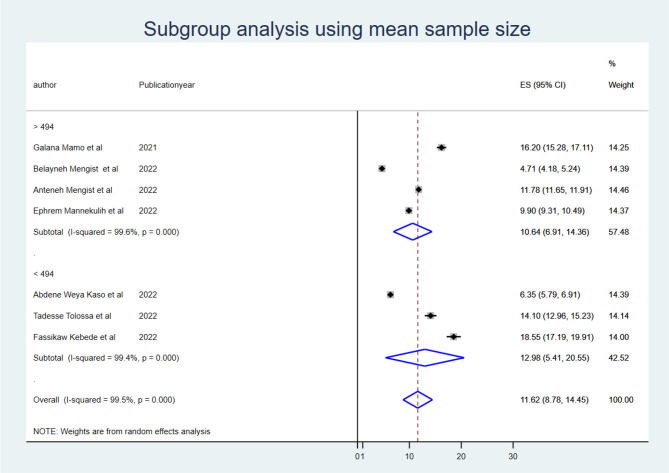
Subgroup analysis using mean sample size.

**Figure 3:**
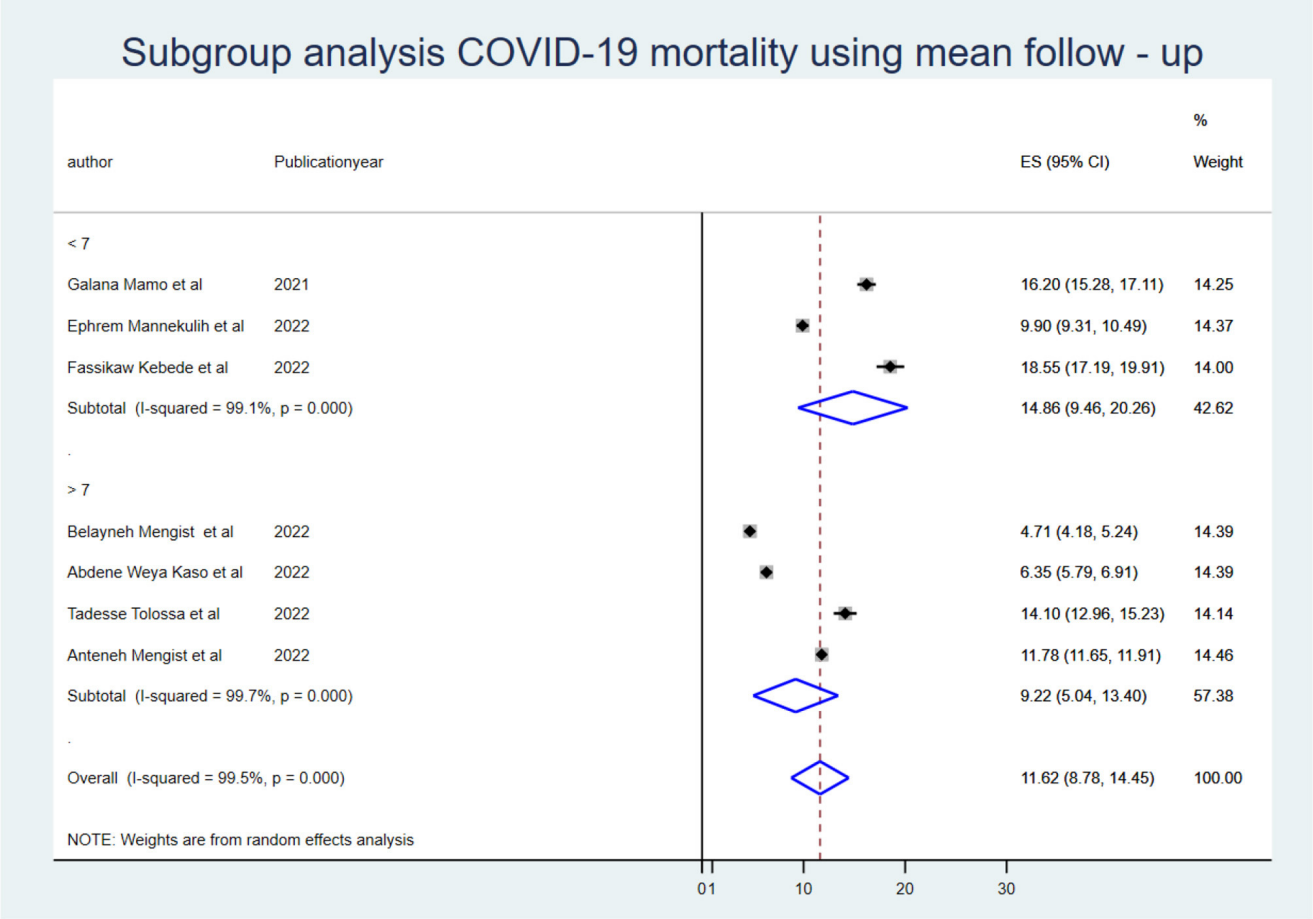
Subgroup analysis using mean follow-up period.

**Figure 4:**
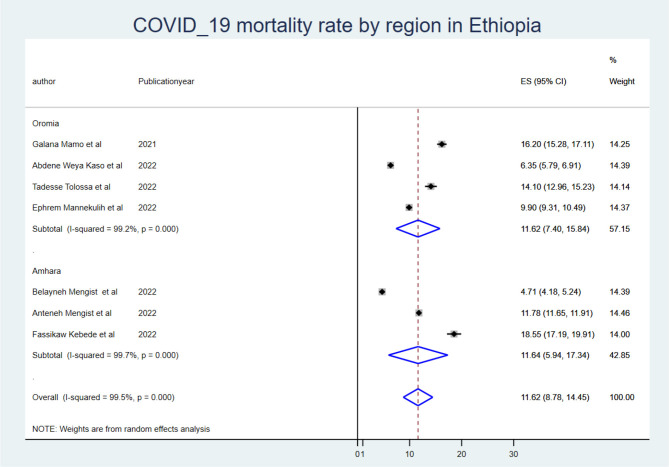
Subgroup analysis using region.

To identify the source of heterogeneity, the subgroup analysis was conducted using region, mean sample size, and mean follow-up period. Hence, the subgroup analysis for sample size was computed using a mean sample size of 494, and heterogeneity was not explained by the sample size (<494) *I*^2^ = 94.8 with a *p*-value of 0.000, and those conducted using a sample size greater than the mean (>494 (*I*^2^: 95.7 with a *p*-value of 0.00) (Figure 2). The mean follow-up period was 7 days, and heterogeneity was not explained by the follow-up period (7 days) *I*^2^ = 96.2 with a *p*-value of 0.000, and studies conducted greater than the mean follow-up days (>7 days) *I*^2^ = 97.6% with a p-value of 0.000 (Figure 3). A subgroup analysis by region was performed, but heterogeneity was not explained by it in the Amhara region (*I*^2^ = 99.0% with a *p*-value of 0.000) or the Oromia region (*I*^2^ = 89.5% with a *p*-value of 0.000) (Figure 4). Finally, the sources of heterogeneity were not identified through the subgroup meta-analyses.

### Meta-regression

In random-effects meta-regression, the year of publication and sample size were used as covariates. According to the findings, sample size (*p* = 0.77) and publication year (*p* = 0.43) did not affect heterogeneity (Table 2).

**Table 2:** The meta regression to detect source of heterogeneity.

**logrr**	**Coeff**	**Std.err**	***t***	***p* > (*t*)**	**95%CI**
Sample size	−5.92	0.00	−0.31	0.77	−0.01, 0.01
Publication year	−0.48	0.54	−0.89	0.43	−1.97, 1.02
Constant	963.68	1091.02	0.88	0.43	−2065.48, 3992.84

### Publication bias (bias detection)

As the number of included articles is < 10, the Egger linear regression test was computed to objectively examine the presence of publication bias. As a result, there was no statistically significant publication bias (*p* = 0.000) (Table 3). As indicated in Table 3, the observed heterogeneity was not due to publication bias.

**Table 3:** The publication bias checking using egger's regression test.

**Std_Eff**	**Coeff**	**Std.err**	***t***	***P* > (*t*)**	**95%CI**
Slope	12.17	1.47	8.30	0.00	8.40, 15.95
Bias	−3.89	3.117	−1.23	0.27	−12.03, 4.25

### Predictors of COVID-19 mortality in Ethiopia

To identify the predictors of COVID-19 mortality, different predictors were considered, among which chronic diseases (e.g., chronic renal disease, diabetes mellitus, and hypertension), HIV infection, and smoking were identified as statistically significant predictors of COVID-19 mortality in Ethiopian COVID-19 patients.

**Figure 5:**
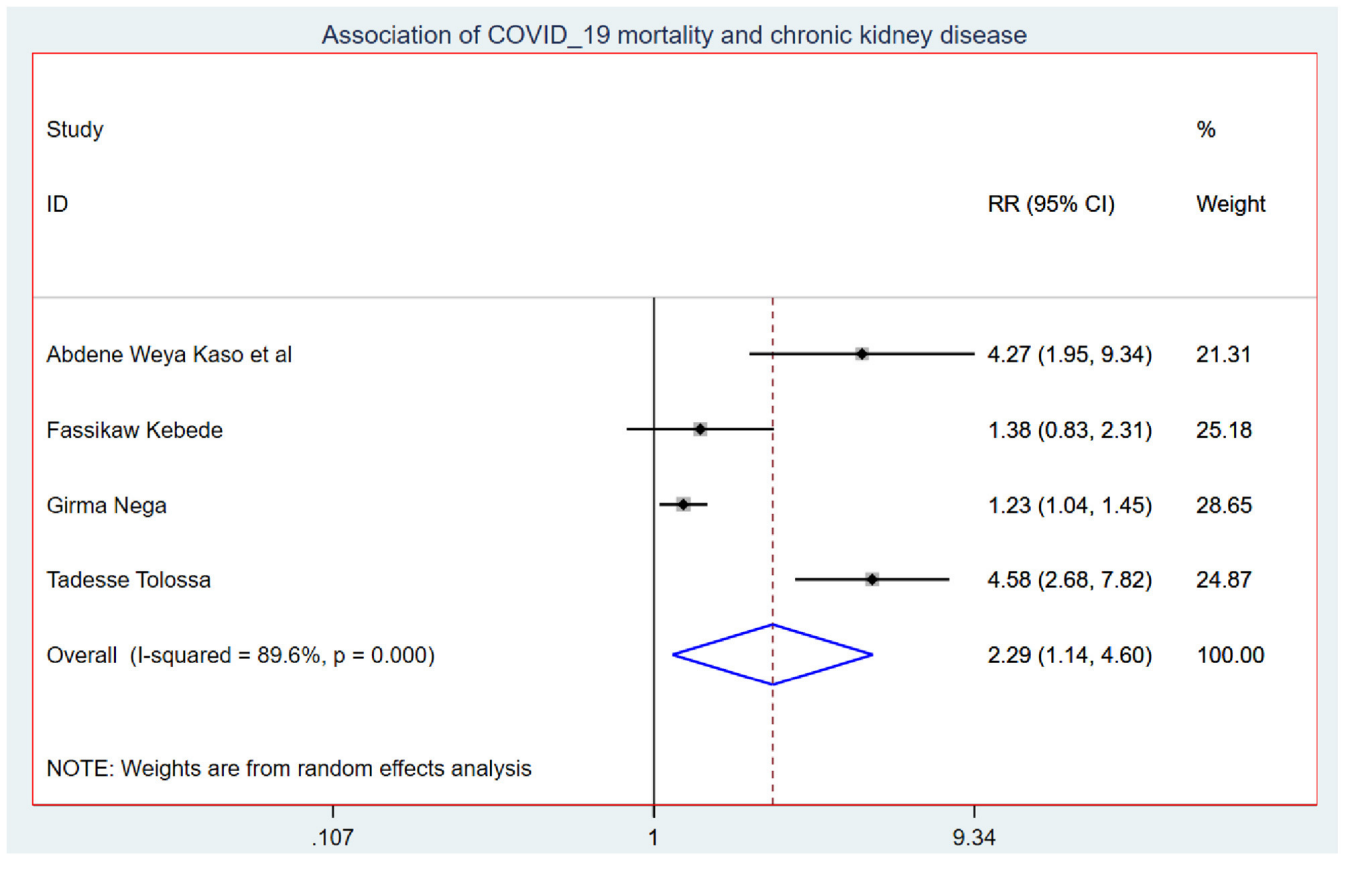
Association of COVID-19 mortality with chronic renal disease in Ethiopia.

**Figure 6:**
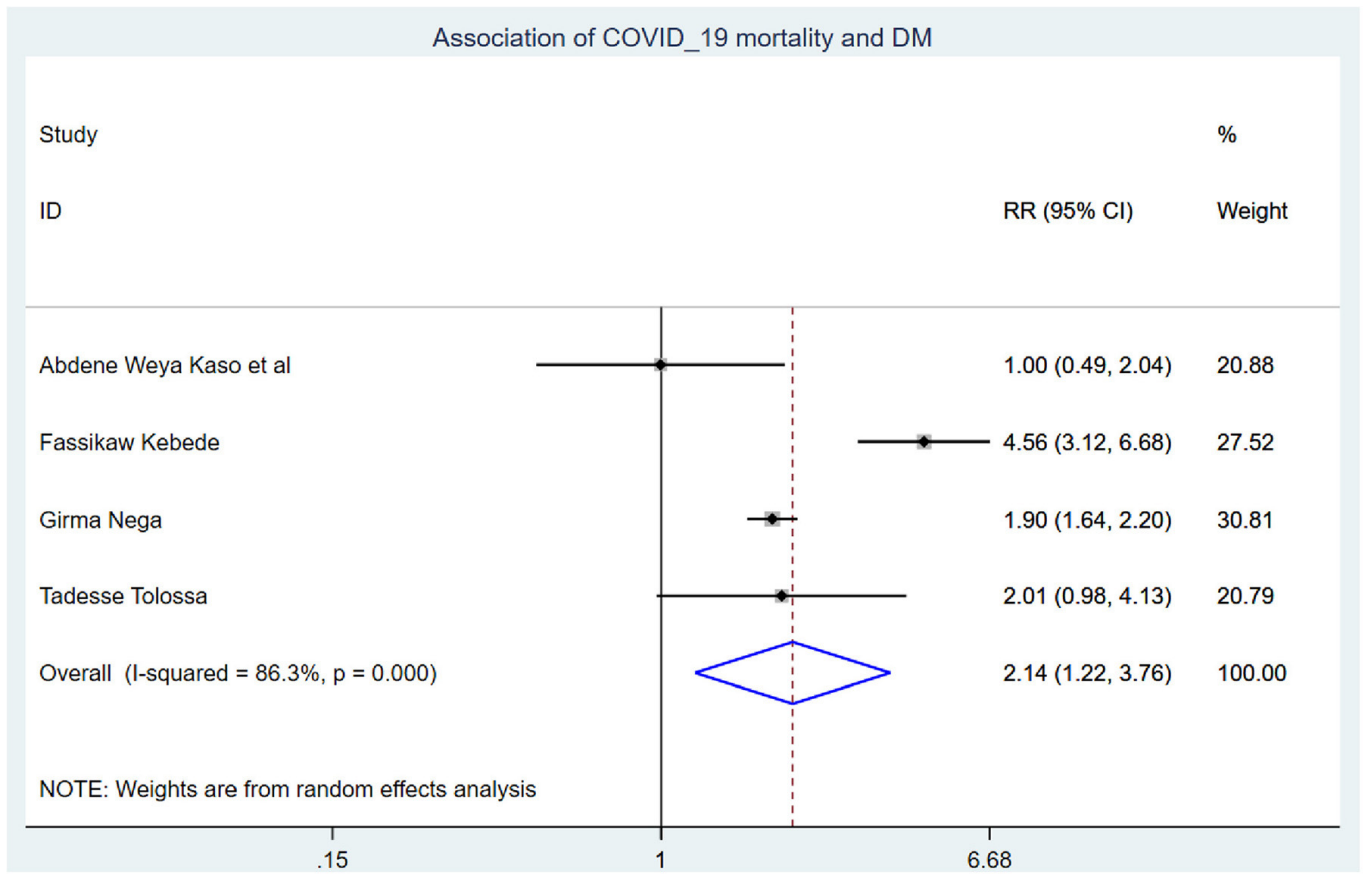
Association of COVID-19 mortality with diabetes mellitus in Ethiopia.

**Figure 7:**
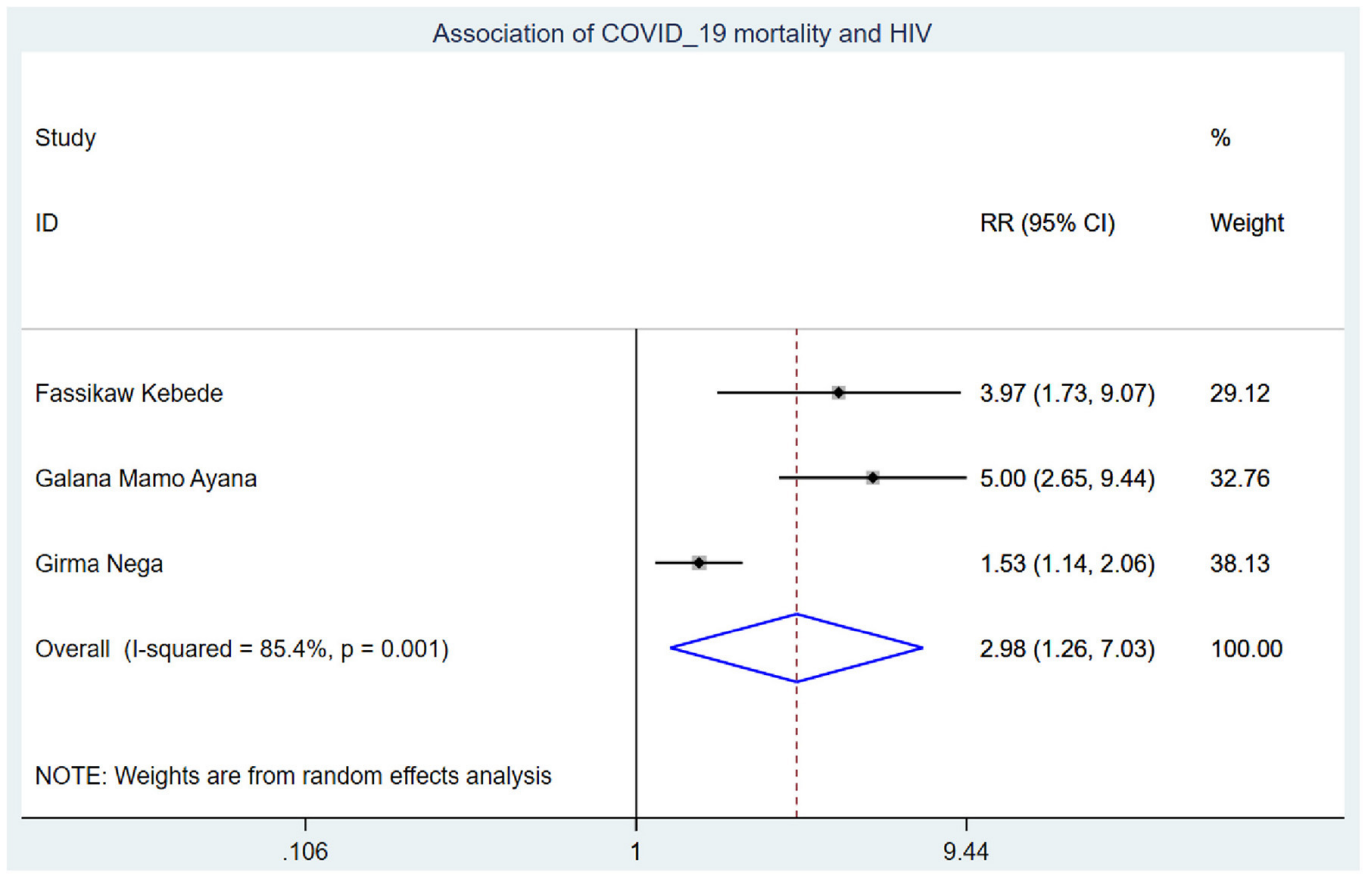
Association of COVID-19 mortality with HIV infection in Ethiopia.

**Figure 8:**
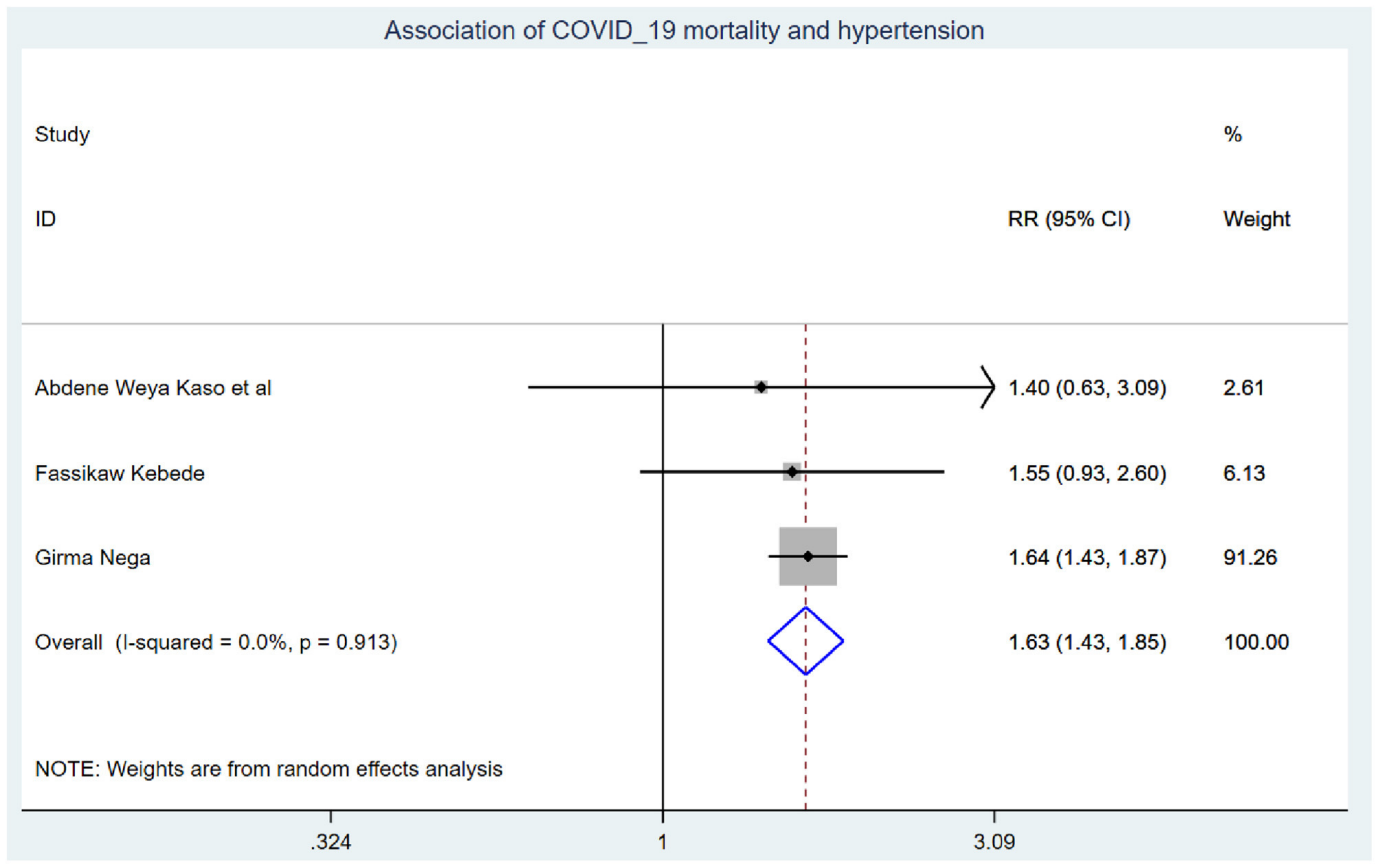
Association of COVID-19 mortality with hypertension in Ethiopia.

**Figure 9:**
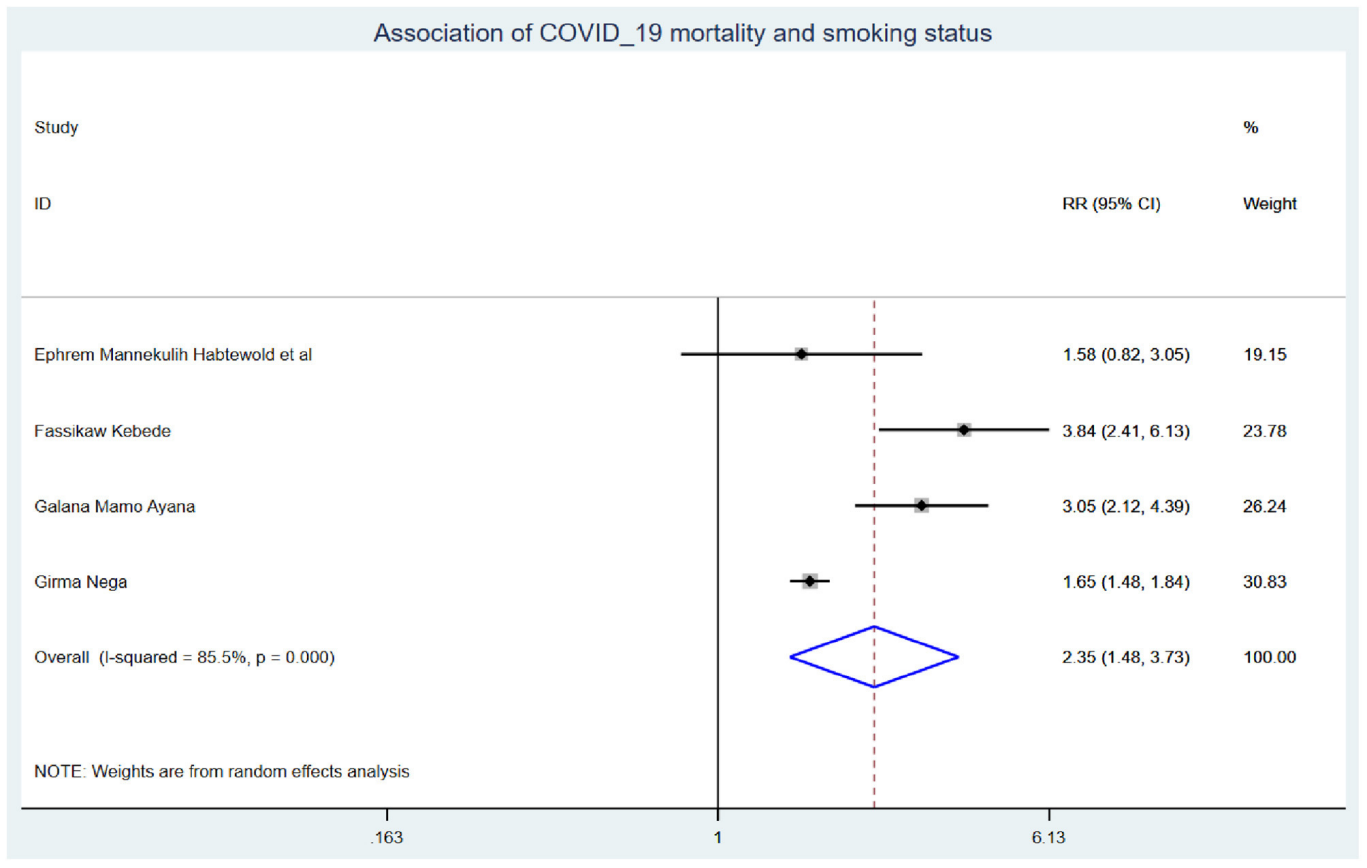
Association of COVID-19 mortality with smoking status in Ethiopia.

Those study participants who had chronic renal disease had 2.29 (95% CI: 1.14, 4.60) times higher chance of mortality when compared with those who were free of chronic kidney disease (Figure 5). COVID-19 patients who had diabetes mellitus had 2.14 (95% CI: 1.22, 3.76) times higher chance of experiencing mortality than patients who were free of diabetes mellitus (Figure 6). The chance of experiencing mortality among patients with HIV infection was 2.98 (95% CI: 1.26, 7.03) times higher than their corresponding counterparts (Figure 7). The risk of COVID-19 mortality among patients with hypertension was 1.63 (95% CI: 1.43, 1.85) times higher than their counterparts (Figure 8). Those patients who were smokers had 2.35 (95% CI: 1.48, 3.73) times higher than their counterparts (Figure 9).

## Discussions

Approximately seven studies with a total of 31,498 study participants were included in this systematic review and meta-analysis in Ethiopia. The eligible studies were from the Amhara and Oromia regions, with the smallest (288) and largest (28,533) sample sizes from the Amhara region. The COVID-19 patients were followed from 3,127 to 243,887 person-days mortality-free observations for 3 to 22 days in the COVID-19 treatment center.

The COVID-19 mortality rate among inpatients was 9.13 (95% CI: 5.38, 12.88) per 1,000 person-days of COVID-19 mortality-free observations in Ethiopia. This is very high for efficient and effective disease control and prevention. This might be because in developing countries such as Ethiopia, the incidence and prevalence of chronic disease have dramatically increased, and chronic diseases make the COVID-19 prognosis worse. This might be the reason for the high onset of COVID-19 mortality in Ethiopia. Hence, the Ethiopian Minister of Health should focus on comprehensive strategies and measures that are built around four components: (1) SARS-CoV-2 strain characteristics, including pathogenicity, infectivity, severity, and mutations; (2) the state of social and economic development, including demographic characteristics, medical resources, material supply, and so on; (3) The level of culture, science, and technology; and (4) The government will prevent and control concepts, social systems, and capacity for social mobilization (34). The Minister of Health should make sure all the necessary medical equipment and medications are used for the treatment of COVID-19 patients. The probability of mortality among COVID-19 patients who had chronic renal disease was 2.29 (95% CI: 1.14, 4.60) times higher when compared with those who were free of chronic renal disease. This finding is supported by a systematic review and meta-analysis conducted in China (35). This could be because CKD patients have higher amounts of pro-inflammatory cytokines, which leads to an increase in oxidative stress, resulting in an inflammatory immunological response. This damages the immune system and makes patients more vulnerable to bacterial and viral coinfection, which could be the major cause of the increased risk of pulmonary inflammation and mortality of COVID-19 patients (36).

COVID-19 patients with diabetes mellitus had a 2.14 (95% CI: 1.22, 3.76) times higher chance of experiencing mortality than patients who were free of diabetes mellitus. This is in line with the study conducted in China (37). This might be due to inflammatory mediators such as IL-1 and TNF that are generated as activated innate immune cells amass in metabolic organs, hastening cell destruction and systemic insulin resistance. Conversely, metabolic difficulties may impede macrophage and lymphocyte immunological function, making the individual susceptible to various disorders and eventually leading to COVID-19 inpatient mortality (38).

The chance of experiencing mortality among patients with HIV infection was 2.98 (95% CI: 1.26, 7.03) times higher than their corresponding counterparts. This finding is supported by the studies conducted previously (39, 40). This could be because HIV patients have higher levels of infectious biomarkers and inflammatory cytokines, which could lead to a large decrease in the number of both helper and suppressor T cells (lymphocytes) in COVID-19 patients (41) as well as persistent immune deterioration manifested by low CD4+ T-cell counts, which was associated with a higher risk of mortality (42). This causes immune activation, respiratory system inflammation, and coagulopathy, making respiration harder and causing inpatients to die (43, 44).

The risk of experiencing COVID-19 mortality among patients who were smokers was 2.35 (95% CI: 1.48, 3.73) times higher than their counterparts. This is in line with the systematic review and meta-analysis conducted previously (45). This could be because the angiotensin II conversion enzyme-2 (ACE2) receptor is utilized for COVID-19 infection, the virus inhibits its normal function, and ACE2 gene expression is higher in smokers. Hence, smoking doubles the vasoconstriction and breathing difficulties in COVID-19 patients, resulting in COVID-19 inpatient mortality (46, 47).

The risk of COVID-19 mortality among patients with hypertension was 1.63 (95% CI: 1.43, 1.85) times higher than their counterparts. This finding is supported by the study (48). This might be because the level of angiotensin-converting enzyme was extremely low among hypertensive patients, which leads to an increased risk of hypertension, myocarditis, and even heart failure and death (49–51).

The included studies were taken from two regions (Amhara and Oromia), which might underestimate or overestimate the figure of the COVID-19 mortality rate in Ethiopia, and this might be taken as the limitation of the study.

## Conclusion and recommendations

In Ethiopian COVID-19 patients, the COVID-19 mortality rate was high despite effective and efficient control of the disease. Chronic renal disease, diabetes mellitus, HIV infection, hypertension, and smoking were identified as predictors of COVID-19 mortality in Ethiopia. Regarding high mortality, healthcare professionals should strengthen their attention to COVID-19 patients admitted with chronic disease (renal, diabetes mellitus, and hypertension), HIV-infected patients, and smokers by providing prioritized care and treatment as well as close follow-ups. COVID-19 patients with comorbidities (chronic renal disease, diabetes mellitus, and hypertension) and HIV should seek healthcare as soon as they experience the clinical presentation of COVID-19. The minister of health should make the treatment center more conducive by overcoming oxygen inaccessibility and integrating treatment for COVID-19 patients who come up with chronic disease comorbidity.

## Data availability statement

The original contributions presented in the study are included in the article/Supplementary material, further inquiries can be directed to the corresponding author.

## Author contributions

Conceptualization, data curation, formal analysis, investigation, methodology, project administration, resources, software, supervision, validation, visualization, writing—original draft, and writing—review and editing: MB and SJ. All authors contributed to the article and approved the submitted version.
